# Bovine Sperm Sexing Alters Sperm Morphokinetics and Subsequent Early Embryonic Development

**DOI:** 10.1038/s41598-020-63077-6

**Published:** 2020-04-10

**Authors:** H. Steele, D. Makri, W. E. Maalouf, S. Reese, S. Kölle

**Affiliations:** 1 School of Medicine, Health Sciences Centre, University College Dublin, Dublin, Ireland; 2 School of Medicine, University of Nottingham, Nottingham, United Kingdom; 3 School of Veterinary Medicine, Institute of Veterinary Anatomy, Histology and Embryology, University of Munich, Munich, Germany

**Keywords:** Embryogenesis, Embryology

## Abstract

In artificial insemination the use of sex-sorted bovine sperm results in reduced conception, the causes of which are only partly understood. Therefore, we set out to investigate the effects of sexing on bovine sperm function and early embryonic development. Computer-assisted semen analysis (CASA) of sperm of the same bulls (n = 5), before and after sexing, demonstrated significantly reduced fast (A) and slow (B) progressively motile sperm (p < 0.05) after sexing. Sexed-sperm also revealed significantly less hyperactivated sperm (p < 0.05). As shown by time-lapse videomicroscopy of *in vitro* produced embryos (n = 360), embryos derived from sexed-sperm displayed significantly increased incidences of arrest at the 4-cell stage (p < 0.05). The relative risk for shrinkage/fusion of blastomeres with subsequent lysis was 1.71 times higher in the embryos derived from sexed-sperm as compared to conventional embryos (p < 0.05) resulting in significantly reduced blastocyst rates (p < 0.001). The relative risk for cleavage was 2.36 times lower in the embryos derived from sex-sorted sperm (p < 0.001). Additionally, sexed-sperm-derived embryos showed reduced survival times (hazard ratio HR = 1.54, p < 0.001) which were bull dependent (p < 0.001). However, the percentage of apoptotic cells was similar to conventional embryos. Furthermore, embryos derived from sexed-sperm were found to reach developmental stages at similar timings as conventional embryos. Our results suggest that reduced conception rates after sexing are due to altered sperm morphokinetics, decreasing the chance of sperm to reach and fertilise the oocyte, and aberrant early embryonic development.

## Introduction

Flow cytometric sorting of spermatozoa is based on differences in the amount of DNA between X and Y bearing sperm, which is detected using the DNA-binding stain Hoechst 33342^1–3^. Due to low sperm numbers, sexed-sperm were primarily first used for *in vitro* fertilization (IVF)^4–6^. Further adaptions at Colorado State University enabled greater sorting efficiency, with the first commercial license for sexed-sperm use in artificial insemination (AI) issued in 2003^7^.

The use of AI has grown to over 130 million artificial inseminations in the dairy industry worldwide annually, with 6% of those being sexed-sperm. In heifers, sexed-sperm accounted for 1.4%, of all registered US Holstein breedings in 2006, 9.5% in 2007 and 17.8% in 2008. In cows in the US, sexed-sperm were used in 0.1% of all breedings in 2006, 0.2% in 2007 and 0.4% in 2008^8^. In 2016, more than 4.5 million straws of sexed semen were processed in the US, over 90% being of dairy sire origin^9^. This increased demand in the dairy industry is because male calves are of low economic value and are associated with a higher risk of dystocia compared to heifer calves^8,10^. Additionally, the use of sexed-sperm in AI enables faster herd expansion, shortened generation intervals and an increased rate of genetic gain^11,12^.

Despite the economic advantages of the use of sexed-semen, its routine use has been limited to date^13,14^. One reason is that the conception rate after the use of sexed-sperm in AI is typically 25% lower across bulls/inseminations compared to conventional sperm^15,16^. Pregnancy rates are 60 to 80% of the conventional sperm range or higher^17,18^, especially with the use of the recently developed SexedULTRA^TM^ technology which included increasing the number of sperm per insemination from 2 to 4 million^19,20^. However, increasing the number of sexed-sperm per insemination dose is unable to fully compensate and restore the reduced conception rate^20–22^. With regards to *in vitro* fertilization (IVF) and *in vitro* production (IVP) of embryos, the use of sexed-sperm results in variable blastocyst rates, ranging from 36% to 52% and subsequent pregnancy rates ranging from 26% to 40%^23,24^. Additionally, the use of sexed-sperm for *in vivo* embryo production in superovulated cows results in considerably fewer recoverable embryos compared to conventional sperm^25,26^. Despite this, calves born of sexed sperm show no discernible abnormalities with 89% born of the correct sex^27^. Further to that, the occurrence of dystocia is decreased from 6% to 2.5%^8^. However, there are indications of a higher incidence of stillbirth in calves of the wrong sex i.e. a male calf when the sperm was sexed for X^28^. Additionally, calves born of *in vitro* produced embryos from reverse X-sorted semen reveal higher cumulative mortality from 90 to 180 d compared to AI offspring. These calves also produce less milk, fat and protein in their first lactation compared to conventional AI offspring^29^.

Sperm-sexing has also been related to a number of molecular alterations such as increased levels of reactive oxygen species (ROS)^30^, increased membrane permeability and reduced intracellular ATP levels, which result in decreased sperm motility, viability and longevity in cattle and horse sperm^31–33^. ROS have been shown to induce DNA fragmentation, leading to impaired membrane fusion and poor fertilisation rates^34^. The sperm chromatin structure assay (SCSA) revealed that bovine sex-sorted spermatozoa have more DNA damage than conventional sperm with reduced chromatin homogeneity and a higher DNA fragmentation index (DFI)^35^. In stallions, DNA fragmentation after sperm sorting is approximately 31%, which is 10% higher than in conventional semen due to oxidative DNA damage which might be induced by dilution, centrifugation, incubation, exposure to DNA stains and laser light^36^. Similarly, decreased progressive motility and increased capacitation after sorting in ram sperm has been observed^37^. Higher numbers of acrosome-reacted sperm post thaw have been reported in sorted bull and ram sperm compared to non-sorted sperm^37,38^. *In vitro* experiments revealed that sexing also affects gameto – maternal interaction by impairing cattle sperm binding to the ciliated cells of tubal explants^39^.

Continuous efforts have been made to improve the technology of flow cytometric sperm sorting. Thus, the pressure used in the flow cytometer has been reduced from 50 to 40 psi, which has improved pregnancy rates^40^. In contrast, reducing the laser power from 150 mW to 50 mW did not have a significant effect on pregnancy rates. The supplementation of antioxidants such as resveratrol in washing and fertilisation media resulted in significantly reduced ROS and increased acrosomal integrity of sperm, leading to increased blastocyst rates and higher embryo quality following IVF^41^. Recently the introduction of SexedULTRA^TM^, a new semen extender designed to provide increased physiological conditions for sperm during sorting and freezing, but not available for the research presented herein, has resulted in significantly more acrosome intact sperm after 3 h at 37 C as well as in significantly increased sperm motility after 24 h compared to conventional sperm^42^.

In spite of these numerous adaptions and modifications, the conception rate after the use of sexed-sperm still remains reduced compared to conventional sperm. This has precluded the widespread adoption of this technology, especially with regards to multiparous cows. Therefore, the aim of our study was to elucidate the effects of sex sorting on sperm function and on subsequent early development of the embryo. In a first step, the sexed-sperm’s ability to reach the oocyte was analysed and compared to conventional sperm through an examination of sperm morphokinetics. In a second step, the developmental competence of embryos derived from sexed-sperm was investigated by assessing the frequency of embryonic arrest, the frequency of shrinkage and/or fusion of embryonic cells, mean time to developmental stage, survival time and survival probability compared to embryos derived from conventional sperm of the same bulls. In a third step, time-dependent embryonic cell numbers and the levels of apoptosis were compared in embryos derived from conventional and sexed sperm of the same bulls.

## Results

### Computer-assisted semen analysis

**Figure 1:**
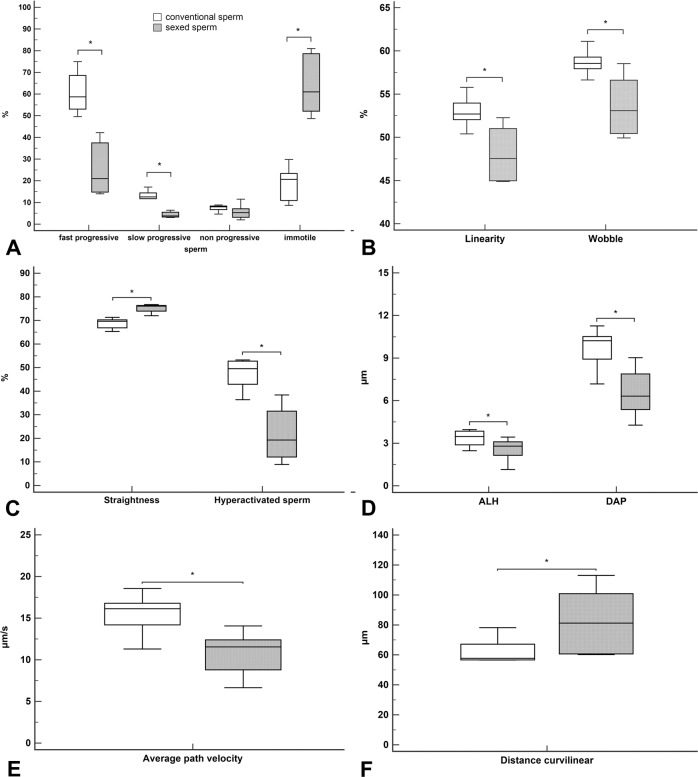
CASA analysis of spermatozoa before and after sexing. **(A**) Percentages of fast and slow progressive sperm are significantly reduced in sex sorted sperm compared to conventional sperm (paired t-test, p < 0.05). The percentage of immotile sperm is significantly increased (paired t-test, p < 0.05). **(B)** The percentage of linearity (LIN) and wobble (WOB) is significantly decreased in sexed sperm compared to conventional sperm (paired t-test, p < 0.05). **(C)** Straightness (STR) is significantly increased in sexed motile sperm as compared to conventional motile sperm (paired t-test, p < 0.05). The percentage of hyperactivated sperm is significantly reduced in sexed-sperm (paired t-tests, p < 0.05). **(D)** The amplitude of lateral head movement (ALH) and distance average path (DAP) are significantly decreased in sexed slowly progressing sperm compared to conventional slowly progressive sperm (Paired t-tests, p < 0.05). **(E)** The average path velocity (VAP) is significantly decreased in sexed slowly progressive sperm compared to conventional slowly progressive sperm (paired t-test, p < 0.05). **(F)** The distance curvilinear (DCL) of the sexed-sperm is significantly increased in slow sperm as compared to conventional sperm (paired t-test, p < 0.05).

CASA analysis of the sperm before and after sexing (n = 5 bulls) demonstrated significantly decreased percentages of sperm with fast (A) and slow (B) progressive motility compared to conventional sperm (25.6% and 4.3% compared to 60.8% and 13.3%, respectively) (Fig. 1A, paired t-tests, p < 0.05). The percentage of sperm with non-progressive motility was similar before and after sexing (Fig. 1A). However, the percentage of immotile sperm was significantly increased after sexing from 18.4% in conventional sperm up to 64.3% in sexed-sperm of the same bull (Fig. 1A, paired t-test, p < 0.05). With regards to progressive sperm, linearity of movement (LIN) and wobble (WOB) were significantly decreased in sexed sperm compared to conventional sperm of the same bull (Fig. 1B, paired t-test, p < 0.05). Straightness (STR) was significantly increased in sexed motile sperm as compared to conventional motile sperm (Fig. 1C, paired t-test, p < 0.05). Furthermore, the percentage of hyperactivated sperm, a motility state characterised by asymmetrical, whip-like movement essential for detachment from the oviductal sperm reservoir, was significantly decreased from 47.3% to 21.7% after sexing (Fig. 1C, paired t-test, p < 0.05). Additionally, the amplitude of lateral head displacements (ALH), which affects overall velocity and straight movement, as well as the distance average path (DAP) were significantly decreased in sexed slowly progressive sperm compared to conventional slowly progressing sperm, which are defined as moving with either VSL < VSL_slow_ or VAP < VAP_slow_ (Fig. 1D, paired t-tests, p < 0.05). Consequently, average path velocity (VAP) was also significantly decreased in sexed progressive sperm (Fig. 1E, paired t-test, p < 0.05). Additionally, the distance curvilinear (DCL) of the sexed sperm was significantly increased in slow sperm after sexing. (Fig. 1F, paired t-test, p < 0.05). The morphokinetic parameters distance straight line (DSL), straight line velocity (VSL), curvilinear velocity (VCL) and beat cross frequency (BCF) were similar before and after sexing.

### Time-lapse videomicroscopy of embryonic development

**Figure 2:**
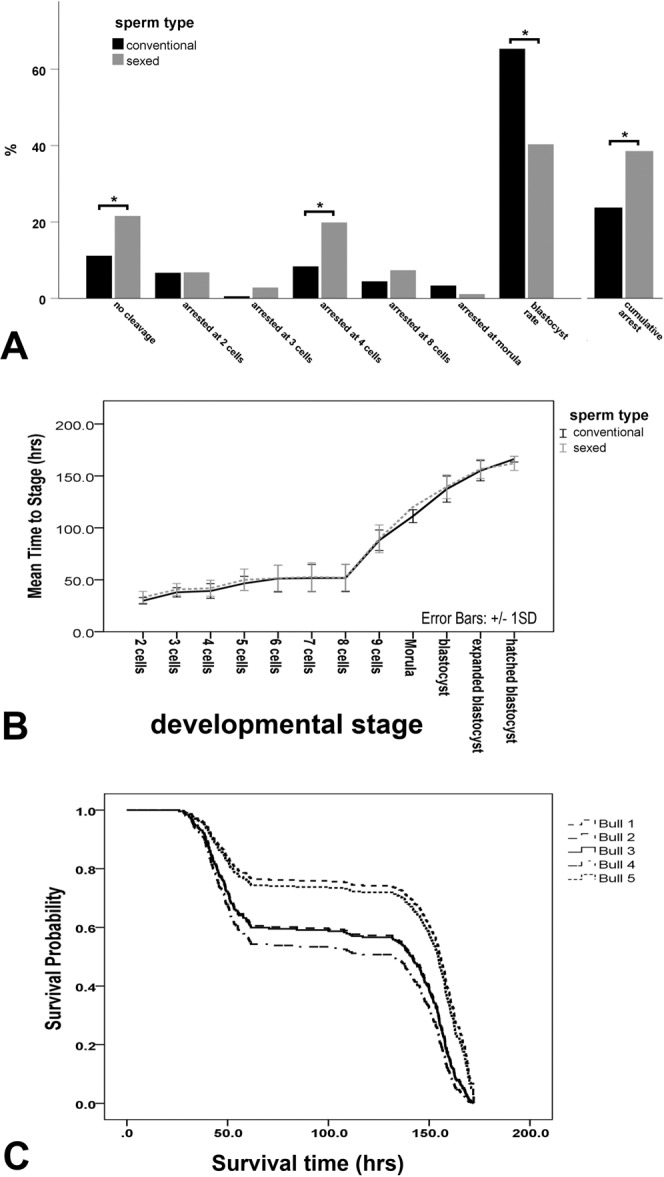
Comparison of embryo arrest, blastocyst rate, mean developmental time and bull effect in sexed and conventional embryos by time-lapse videomicroscopy. **(A)** The percentage of unfertilized oocytes and arrested zygotes is significantly higher when using sexed-sperm compared to conventional sperm. Sexed-sperm derived 4-cell embryos and blastocysts reveal significantly increased embryo arrest compared to conventional embryos (Fisher’s exact test, p < 0.05). The cumulative arrest of the 2 cell embryo to blastocyst stage was significantly increased in embryos derived from sexed-sperm compared to conventional embryos (Chi-Square Test, p < 0.05) **(B)** There was no significant difference in average timing of embryonic development before and after sexing (p > 0.05, Mann-Whitney U-test). **(C)** The individual sire had a significant effect on embryo survival time (Cox proportional-hazards regression, p < 0.001).

**Figure 3:**
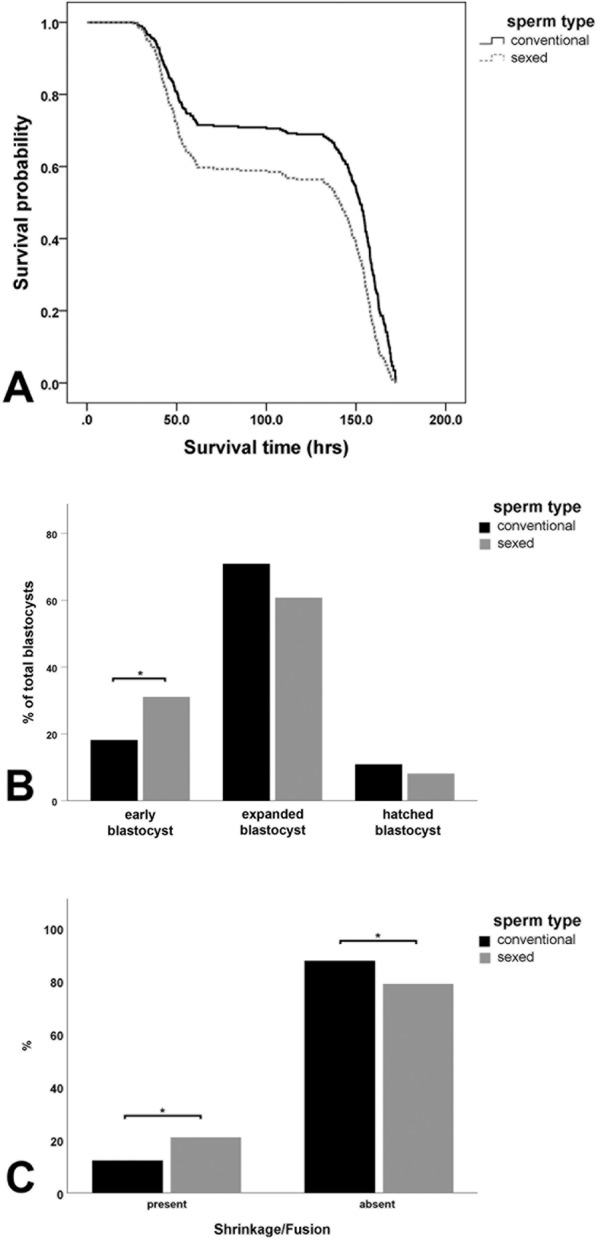
Comparison of survival time, blastocyst development and the presence of cellular shrinkage/fusion in sexed and conventional embryos by time-lapse videomicroscopy. **(A)** Sexing had a significant negative effect on embryo survival time (Cox proportional-hazards regression, p < 0.001). **(B)** When comparing the percentage of early blastocysts, expanded blastocysts and hatched blastocysts in conventional and sexed sperm derived embryos on day 7 (165 h after insemination), significantly more sexed-sperm derived embryos were at the blastocyst stage and had not developed further to the expanded or hatched blastocyst (Chi-square test, p < 0.05), **(C)** Sexed -sperm derived embryos revealed significantly more shrinkage/fusion events compared to conventional embryos (Chi-square Test, p < 0.05).

Time-lapse video microscopy of embryo culture of 360 embryos using the semen from 5 bulls before and after sexing revealed that the use of sexed-sperm resulted in significantly increased percentages (from 10.6% to 17.7%) of unfertilized oocytes and arrested zygotes, respectively (Fisher’s exact test, p < 0.05, Fig. 2A). In the bovine, unfertilized oocytes and arrested zygotes cannot be discriminated due to the dark cytoplasm masking the presence or absence of pronuclei. This is in contrast to human and mouse zygotes where the apposition of the pronuclei can be visualized. Furthermore, embryos derived from sex-sorted sperm were significantly more likely to arrest at the 4-cell stage (Fisher’s exact test, p < 0.05, Fig. 2A). Moreover, the blastocyst rate was significantly decreased from 65% to 39% in sexed embryos (Fisher’s exact test, p < 0.001, Fig. 2A). Overall, the cumulative arrest of the embryos from the 2-cell stage to the blastocyst was significantly higher in embryos derived from sexed-sperm compared to embryos derived from conventional sperm (Fig. 2A, p < 0.05, Chi-Square test). The relative risk for cleavage was 2.36 times lower in the embryos derived from sexed sperm (p < 0.001, Chi-Square test). The numbers and percentages of embryos at each available developmental stage are outlined in Table 1 of the supplemental data. The mean developmental time to a certain embryonic stage was not signifantly different in embryos derived from sexed-sperm compared to embryos derived from conventional sperm (Mann-Whitney U-test, Fig. 2B). In regard to survival probability of embryos derived from sexed-sperm, a highly significant individual sire effect was found on embryo survival time (Cox proportional hazards regression, p < 0.001, Fig. 2C) highlighting that the survival time of embryos after sex sorting is bull-specific. When comparing the survival time in the two different groups sexed-sperm-derived embryos revealed a significantly decreased survival time compared to conventionally derived embryos (Cox proportional hazards regression, p < 0.001, Fig. 3A). The Cox regression (or proportional hazards regression) used for statistical analysis in this experiment is a method for investigating the effect of several variables upon the time a specified event will happen. In the actual context of an outcome such as embryonic death, this is known as Cox regression for survival analysis (Fig. 2C).

**Figure 4:**
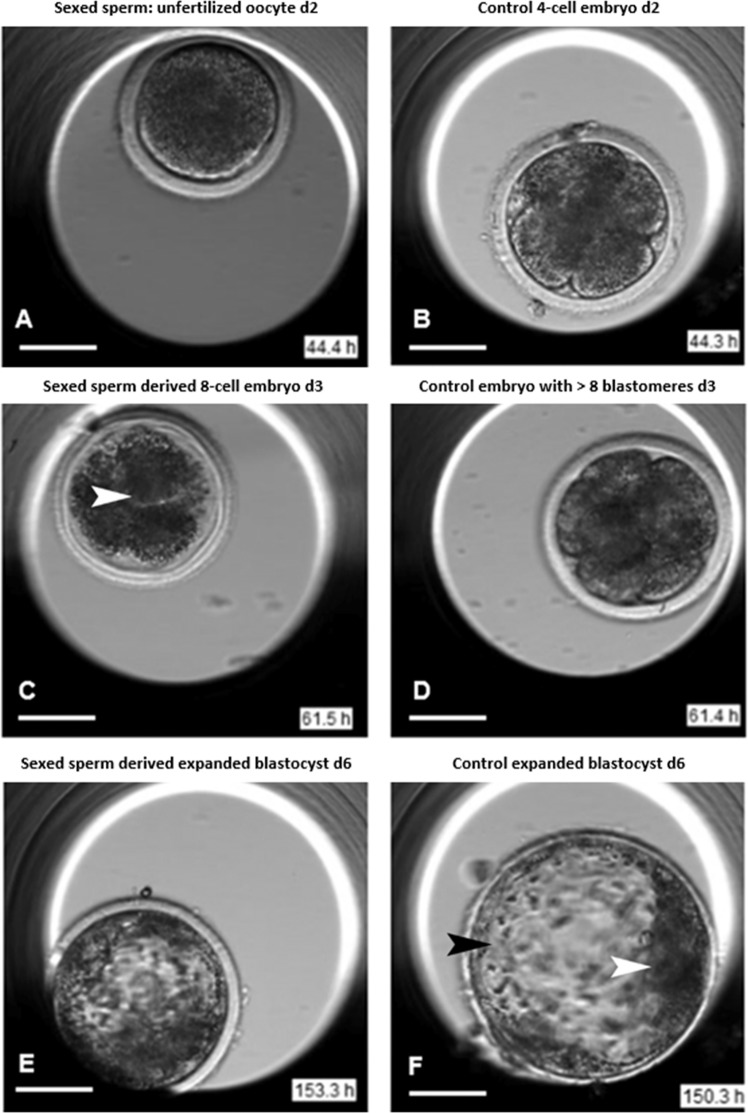
Time-lapse videomicroscopy of sexed and conventional early embryos. **(A)** Unfertilized oocyte or zygote displaying cleavage failure on day 2 (44.4 h) after insemination. **(B)** Control 4-cell embryo on day 2 (44.3 h after insemination). **(C)** Sexed sperm derived embryo on day 3 (61.5 h after insemination) displaying blastomere lysis (arrow). **(D)** Control embryo on day 3 (61.4 h) after insemination revealing >8 blastomeres. **(E)** Sexed sperm derived expanding blastocyst on day 6 (153.3 h after insemination). **(F)** Conventional sperm derived expanded blastocyst with trophectoderm cells (black arrowhead) and inner cell mass (ICM, white arrowhead) on day 6 (150.3 h after insemination).

When comparing the percentage of early blastocysts, expanded blastocysts and hatched blastocysts in conventional and sexed-sperm-derived embryos on day 7 (156 h after insemination), significantly more embryos derived from sexed-sperm were in the blastocyst stage and had not developed further to the expanded or hatched blastocyst stage (Chi-square Test, p < 0.05, Fig. 3B) resulting in fewer expanded and hatched blastocysts. The overall risk of cleavage failure is 2.36 times higher in sexed sperm-derived embryos (Fig. 4A) compared to conventional embryos (Fig. 4B). Movie 1 shows an unfertilized oocyte after incubation with sexed sperm or a zygote which is to cleave and undergo further development. In addition, embryos derived from sexed-sperm were significantly more likely to undergo a lysis/shrinkage/fusion event and subsequent developmental arrest (Chi-square Test, p < 0.05, Figs. 3C and 4C), with the relative risk being 1.71 times higher as compared to the conventionally derived group (Figs. 3C and 4D). Movie 2 shows an embryo derived from sexed-sperm undergoing blastomere lysis. However, if a sexed sperm-derived-embryo reaches the blastocyst stage the cell morphology and developmental timing is similar to an embryo derived from conventional sperm (Fig. 4E,F, black arrowhead: trophectoderm cells, white arrowhead: inner cell mass (ICM), Movies 3 and 4).

### Total cell number and percentage of apoptotic cells

**Figure 5:**
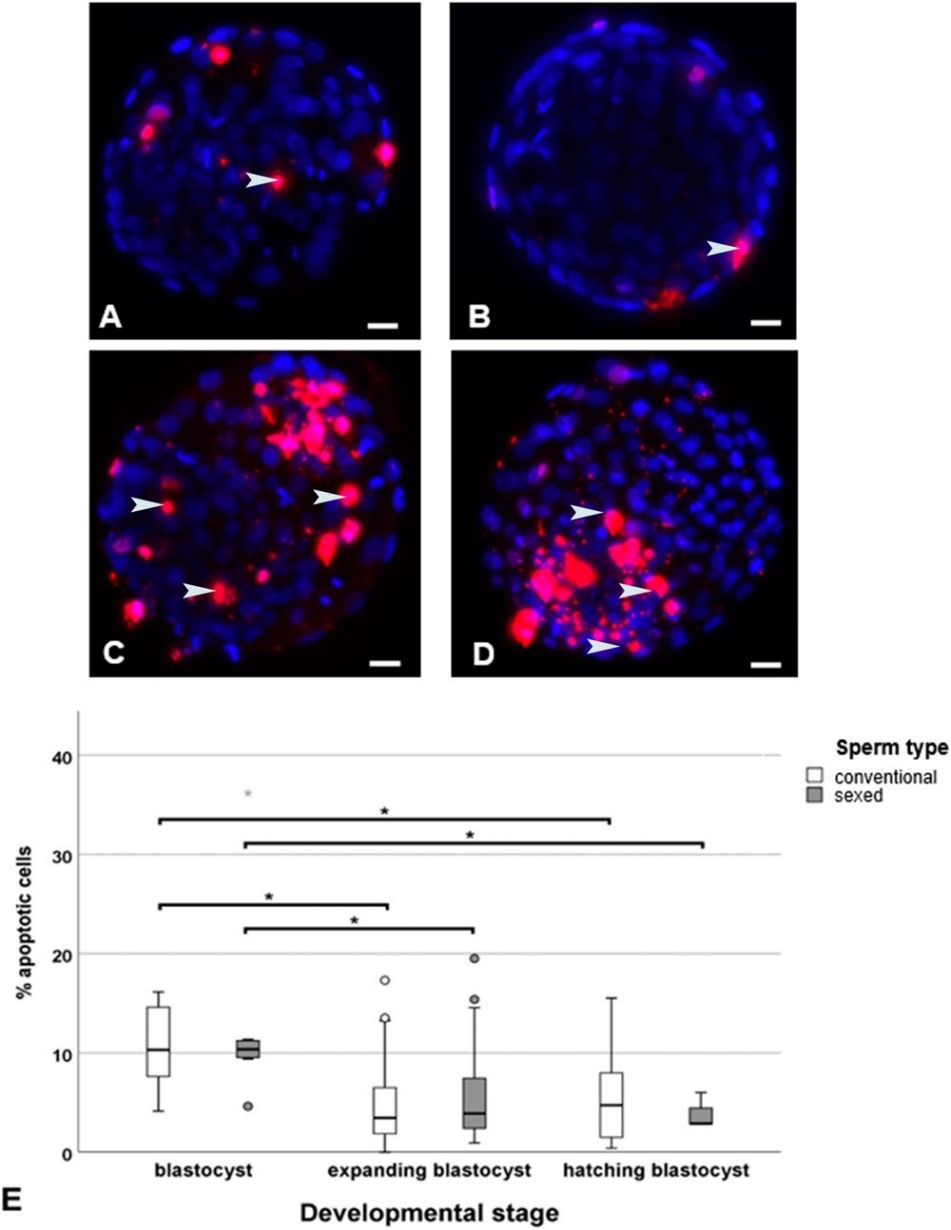
Cell numbers and apoptosis in sexed-sperm derived embryos vs conventional embryos. **(A)** Sexed sperm derived blastocyst **(B)** Conventional blastocyst **(C)** Sexed sperm derived expanding blastocyst **(D)** Conventional expanding blastocyst. Staining was performed with DAPI (blue staining), a DNA stain for total cell counting, and TMR red (red staining) for the determination of apoptosis (arrowheads). **(E)** Apoptosis in embryos derived from sexed-sperm was not significantly altered compared to embryos derived from conventional sperm. When comparing different stages of embryonic development the percentage of apoptotic cells was significantly higher in blastocysts compared to expanding and hatching blastocysts in both groups (generalized estimating equation, p < 0.05).

A total of 164 blastocysts were analysed for total cell number. No significant difference was detected between the blastocysts derived from sex-sorted sperm compared to conventional sperm derived embryos (Fig. 5A,B, blue staining). Similarly, the percentage of apoptotic cells was similar for sexed-sperm-derived expanding blastocysts (7.2%) compared to conventional sperm derived expanding blastocysts (7.1%) (Fig. 5C–E, red staining, arrowheads). Thus, sperm sexing had no significant influence on the rate of apoptosis (generalized estimating equation, p = 0.114). When comparing different developmental stages, the percentage of apoptotic cells was significantly higher in blastocysts (11.8%) compared to expanding blastocysts, (5.1%, Fig. 5E, generalized estimating equation, p < 0.001) and hatching blastocysts (5.3%, Fig. 5E, generalized estimating equation, p < 0.001) in both embryos derived from sorted and conventional sperm. Red dots (regular small size) which were not co-localized with nuclei were regarded as unspecific staining and were not included in the counting (Fig. 5C,D). In blastocysts, typically the whole nucleus was stained red indicating apoptosis. Contrary to this in expanded blastocysts, spotted and irregular labelling was observed in single nuclei indicating the start of apoptosis in these nuclei, which were not included in the analysis. There were no significant differences in the percentage of apoptotic cells in expanding and hatching blastocysts (5.1% and 5.3%, respectively, generalized estimating equation, p = 0.771).

## Discussion

The present study is the first to compare all parameters of sperm morphokinetics before and after sexing coupled with uninterrupted time-lapse embryo videomicroscopy in cattle, providing the first in depth analysis of early sexed-sperm derived embryonic development. It is the first study comparing the frequency of embryonic arrest, embryonic shrinkage, mean developmental time and survival probability in conventional and sexed sperm-derived embryos. Additionally, it is the first study to compare programmed cell death (apoptosis) within different stages of embryo development before and after sexing.

Sperm parameters were examined through CASA, which allowed for the comparison of sperm kinematics in the same bulls before and after sexing in an unbiased manner with high precision and accuracy^43,44^. Quantitative analyses revealed highly significant alterations, in both sperm motility and sperm morphokinetics compared to conventional sperm. Thus, after sex sorting, there was a significantly reduced percentage of fast (A) and slow (B) progressively motile sperm compared to non-sorted sperm. Reduced motility results in less sperm arriving at the site of fertilization. Although movement of spermatozoa is supported by smooth muscle contraction of the female genital tract^45^, high motility is pivotal to allow the spermatozoa to escape from the strong turbulences near the cilia of the tubal wall and to reach the middle of the lumen of the fallopian tube^46–48^. In addition, sexed-sperm also revealed significantly reduced percentages of hyperactivated sperm. Hyperactivation is characterized by high-amplitude, asymmetrical flagellar bending^49^ and is important for the sperm to detach from the tubal sperm reservoir, to migrate through the oviduct and to reach the oocyte^50^. Hyperactivation also allows the sperm to penetrate the cumulus oophorous^51^. Furthermore, in sexed-sperm, there was a significantly increased percentages of immotile (D) sperm, meaning that less sperm are able to migrate through the reproductive tract. This also implies that during the journey of the spermatozoa within the female genital tract more sperm are selected out at the barriers which include the cervix and the utero-tubal junction^52^. Overall the decreased motility and hyperactivation together with the high percentage of immotile sperm result in fewer sperm bound in the sperm reservoir, which maintains sperm vitality and fertilizing capacity^53,54^. This is significant given that the number of sexed-sperm is already reduced in the insemination dose compared to the conventional sperm insemination dose^55^.

Besides reduced motility and hyperactivation, sexed-sperm show significantly decreased average pathway velocity (VAP) and distance average path (DAP) implying that their progress through the oviduct *in vivo* is significantly slower. This might result in miss-timed sperm-oocyte interaction so that spermatozoa reach the waiting oocyte outside the fertilisation window. This is supported by the increased conception rate of sexed-sperm associated with the use of split-time artificial insemination (STAI)^56^, highlighting the need for precise sperm-oocyte timing when using sorted sperm. However, the accuracy of oestrus detection as part of the STAI regime is a confounding factor on the pregnancy rates associated with sorted sperm. Due to the variability of individual systems in terms of accuracy and feasibility of oestrus detection, the use of sexed semen is only advisable in cows expressing oestrus^20^. Sexed-sperm also displayed significantly reduced amplitude of lateral head displacement (ALH) implying that they display a straighter and slower forward motility than their conventional counterparts. Correlated to this finding, is the significantly increased straightness (STR) linked to reduced wobble (WOB) of sexed sperm which highlights that they are moving with symmetrical motility compared to the conventional sperm which are moving with asymmetrical motility due to hyperactivation. These altered patterns in sperm kinematics add to reduced numbers of sperm reaching the site of fertilization. This also means that under *in vivo* conditions the sexed-sperm may be less likely to successfully penetrate through the matrix of the cumulus-oocyte-complex and successfully fertilise the oocyte. Further to that, the paucity of competent sperm might cause a delay of fertilization resulting in the presence of an older, less competent oocyte at the time of fertilization.

In summary, these results indicate that sexed-sperm kinetics are highly discordant to the kinetics of conventional sperm. This points to a highly altered motility pattern *in vivo*, which results in decreased numbers of sperm with reduced fertilizing capacity, which may be key target areas for post-sorting sperm remediation.

The sex sorting process does not only impair sperm motility and sperm kinetics but also compromises subsequent early embryonic development. Using time-lapse videomicroscopy we were able to continuously monitor embryos derived from sexed and conventional semen from the zygote to the blastocyst stage. Our results show that the use of sexed-sperm results in higher percentages of unfertilized oocytes and arrested zygotes In cattle, unfertilized oocytes and arrested zygotes cannot be discriminated due to the dark cytoplasm of the oocyte. Furthermore, embryos derived from sexed-sperm revealed a significantly higher probability to arrest at the zygote and 4-cell stage. Arrest at the zygote stage implies that after apposition of the male and female pronuclei subsequent mitotic events fail to take place. Arrest at the 4-cell stage is associated with failure of the transition from totipotency with complete differentiation potential of the cells to pluripotency^57^. The high probability of arrest is accompanied by reduced cleavage of embryos derived from sexed-sperm. Our results are confirmed by Bermejo-Alvarez *et al*. (2010) who observed that the use of sorted sperm significantly delays the onset of the first cleavage^58^. Higher percentages of arrested embryos in combination with reduced cleavage results in decreased blastocyst rates and thus reduced pregnancy rates *in vivo*. The data obtained also suggests that sexed semen is relatively less efficacious *in vitro* than *in vivo*. This is supported by studies that reported that sorted sperm produce fewer blastocysts via IVF than non-sorted sperm^59,60^. Further to that, our study provides the first evidence that sexed-sperm derived embryos have reduced developmental competence linked to aberrant development characterised by increased embryo arrest and increased incidences of shrinkage/fusion events.

The final consequence is the known reduced conception rate when using sexed-sperm. This correlates with findings which report an increase up to 59% in recovered degenerate embryos recovered after superovulation and insemination with sexed sperm compared to 24% when using conventional sperm^61^. Possible explanations for the compromised early embryonic development are a) Hoechst 33342 binding which slows down DNA and RNA synthesis and b) polyspermy mediated by inefficient triggering of the fast block to polyspermy by sexed-sperm. Whilst Hoechst 33342 was the final optimization step in allowing fluorescence activated cell sorting of viable sperm with research suggesting its non-toxicity^3,62^, there have been previous reports of its mutagenicity^63^. Interestingly, in the aforementioned study, Hoechst 33342 was cleared by two mechanisms, first a rapid phase with the remaining intercalated dye cleared by cellular division pointing to possible mechanisms for the shrinkage/fusion events found in this study. The observed increased incidences of cellular arrest and shrinkage/fusion events in the embryos derived from sexed-sperm exert significant deleterious effects on the average survival time of embryos. Thus, embryo survival time using sex sorted sperm was significantly decreased compared to their conventional counterparts. Interestingly, this appears to be bull dependent, which correlates with reported high individual sire variability in IVP of bovine embryos^64,65^. This highlights that sperm of individual bulls are affected by sexing in different, bull-specific ways^66^.

When comparing the timing of cell divisions of embryos derived from sexed-sperm to that of conventionally derived embryos, no statistical difference was discernible, which was independent from the sex. In contrast, male human embryos have been found to develop at a significantly faster rate than female embryos^67,68^. Moreover, there was no increase in the percentage of apoptotic cells in embryos derived from sexed-sperm compared to the conventional sperm derived embryos, suggesting that sperm sexing does not cause downstream cell death beyond the blastocyst stage. This is supported by the fact that *in vivo* produced sexed -sperm derived blastocysts have been shown to have similar frozen thawed pregnancy rates as conventional sperm derived blastocysts^69^. In summary, our studies have shown that sexing of bovine sperm results in impaired motility and altered morphokinetics of spermatozoa and compromised early embryonic development. Our results suggest that the effect of sperm sexing is additive, with multiple processes affected. Thus, in regard to sperm morphokinetics, sex sorting leads to increased numbers of immotile sperm and decreased numbers of progressive and hyperactivated sperm, implying that fewer sperm can detach from the tubal sperm reservoir and can successfully migrate through the female genital tract. Furthermore, sperm movement is altered with decreased lateral head displacement, distance average path and average path velocity which negatively affects overall velocity. As a consequence, not only do fewer sperm reach the oocyte but they also may arrive later causing them to meet an older oocyte with reduced developmental capacity. In regard to the early embryo, the reduced conception rates seen in field studies can be related to altered embryonic development, specifically decreased capacity for cleavage and for development to the expanded blastocyst, increased embryonic arrest, increased embryonic cell shrinkage, increased embryonic cell fusion and reduced survival time. These alterations may be due to epigenetic changes, which warrants further investigation. The results of this study also emphasize that the process of sex-sorting affects sperm in a manner which persists beyond fertilization and highlights the fact that sperm integrity plays a major role in embryo quality. Functional knowledge of the post-sorting alterations in sperm integrity and early embryonic development are pivotal in developing cost-effective remediation strategies to improve conception rates *in vivo* which will enable widespread adoption of this technology.

## Materials and Methods

### Ethics

This project was exempt (AREC-E-18-46-Koelle) from internal animal care and use approval, as samples were collected from post-mortem animals according to the Animal Research Ethics Committee at University College Dublin.

### Spermatozoa

Cryopreserved semen from 10 Holstein Friesian bulls (aged 3-6 years) before and after sexing were obtained from Rinderzucht Schleswig-Holstein, Neumuenster, Germany. Sperm were sorted using the routinely used flow cytometric sperm sexing technology, which was developed by Johnson *et al*., in the United States Department of Agriculture^2^ (referred to the Beltsville sperm sexing Technology^70^, patented in 1991). The patent for non-human animals was licensed by the USDA to XY Inc, which commercialized the technology of sperm sexing, and was acquired by Sexing Technologies (Navasota, TX, USA) in 2007.

### Computer assisted semen analysis (CASA)

Cryopreserved semen from 5 Holstein Friesian bulls (aged 3-6 years) before and after sexing (n = 5) were analysed. Quantitative parameters of sperm motility were determined using a computer-assisted semen analysis (CASA) instrument; Hamilton Thorne, Beverly, MA) and the software IVOS II, version 1.10 (Hamilton Thorne, Beverly, MA). Sperm were thawed at 38 °C for 10 s prior to analysis. Median values of each of the kinematic parameters were obtained for each sample. Kinematic parameters measured included curvilinear velocity (VCL), straight-line velocity (VSL), average path velocity (VAP), amplitude of the lateral head displacement of sperm (ALH), beat-cross frequency (BCF), linearity (LIN = 100% × VSL/VCL), and straightness (STR = 100% × VSL/VAP). Sperm were counted as motile with any type of movement and progressively motile when VAP > 50 μm/sec and STR > 50%. Hyperactive sperm were identified with the ‘sort fraction’ function of the Hamilton Thorne analyser using the following criteria: Fractal dimension (FDM): FDM = log (n)/ [log (n) + log (d/L)] where: d = maximum DSL (straight line distance) for track, L = total DCL (curvilinear distance) for a track, n = number of points in a track – 1 (one less than the number of points in a track). If FDM was> 1.3, the track was considered fractal^71^. Further to that sperm motility was characterized as A, B, C, and D according to the following criteria: A > 25 μm/s (fast progressive sperm), B 5-25 μm/s (slow progressive sperm), C 0-5 μm/s (non-progressive sperm) and D 0 μm/sec (immotile sperm). The CASA was parameterized as follows: frames acquired – 100, frame rate – 60 Hz, minimum cell size – 50 pixels, straightness threshold – 80%, average pathway velocity (VAP) cut-off *–* 25 μm/s, straight line velocity (VSL) cut-off – 20 μm/s, cell intensity – 80, static elongation – 11 to 80, magnification 1.89.

### Oocyte collection and maturation

Ovaries from cows (n = 173) were collected immediately after slaughter from the abattoir (Foyle Meats, Melton Mowbray, UK). Specimens were put into a thermos flask in Dulbecco’s PBS (Sigma-Aldrich, USA) at 37 °C and transported to the laboratory within 1 hour. Cumulus-oocyte complexes (COCs) were aspirated from visible follicles (2-10 mm in diameter) using a 25-gauge needle and syringe. Only oocytes with three or more layers of cumulus cells were used for *in vitro* maturation. Groups of 45 COCs were washed three times in search media (S199, IVF Bioscience, Falmouth, UK) and then cultured in 500 μl of BO IVM culture medium (IVF Bioscience, Falmouth, UK) in 4-well dishes (Nunclon, Roskilde, Denmark) at 38.8 °C and 6% CO_2_ for 24 h (IVF Bioscience, Falmouth, UK).

### Preparation of sperm for IVF

Five different bulls (breed: Holstein-Friesian, age: 3-6 years) were used for analysis, using semen from each individual bull analysed before and after sexing (n = 5). Frozen semen was thawed at 38 °C for 10 s. The sperm were then washed twice in 2 ml of BO-SemenPrep (IVF Bioscience, Falmouth, United Kingdom) and centrifuged at 200 g for 5 min. The pelleted sperm were re-suspended in 350 μl of BO-IVF (IVF Bioscience, Falmouth, UK). Sperm concentration was calculated using the Neubauer Chamber.

### In-vitro fertilization (IVF)

Five IVF cycles were performed (n = 360 oocytes) using five bulls (n = 5), with semen from each individual bull before and after sexing. Following IVM oocytes were washed three times in oocyte wash medium (IVF Bioscience, Falmouth, UK and once with BO-IVF medium IVF Bioscience, Falmouth, UK). The oocytes were then transferred to 400 μl of BO-IVF medium (IVF Bioscience, Falmouth, United Kingdom) with sexed or conventional spermatozoa, at a final concentration of 10^6^ spermatozoa mL^−1^, and were cultured in 4-well dishes (Nunclon, Roskilde, Denmark) at 38.8 °C and 6% CO_2_ in humidified air overnight.

### In-vitro embryo production (IVP) using time-lapse videomicroscopy

The presumptive zygotes (n = 360) were washed in oocyte wash medium and vortexed in 1 ml of oocyte wash medium to remove the cumulus cells. Specimens were then washed three times in oocyte wash medium and once in BO-IVC (IVF Bioscience, Falmouth, UK) medium before being put in individual wells in time-lapse dishes (Parallabs, United Kingdom) filled with BO-IVC medium (IVF Bioscience, Falmouth, UK). Specimens were then placed in an EmbryoScope time-lapse incubator (Vitrolife, Gothenburg, Sweden) at 38.8 °C and 6% CO_2_ in 6% O_2_. Each dish contained 12 individual wells containing 25 μl of BO-IVC medium per well overlaid with 1.4 ml of BO-Oil (IVF Bioscience, Falmouth, United Kingdom). Cleavage rate was recorded at 48 hrs post insemination (psi) and blastocyst development was recorded at 165 hrs psi. Cell stage at time of embryo arrest, time-to-two cell, time-to-four cell, time-to-morula, time-to-blastocyst, time-to-expanded blastocyst and time-to-hatching blastocyst were recorded by the Embryo-Scope software (Vitrolife, Göteborg, Sweden)^72^. Early blastocysts were identified by the formation of the blastocoel, expanded blastocysts by thinning of the zona pellucida of >50%, and hatched blastocysts by absence of the zona pellucida. Shrinkage/fusion events were annotated using the full time-lapse video for each embryo, with a shrinkage/fusion event being defined as an event where two or more blastomeres fuse together and lyse resulting in cellular arrest. Blastocyst development was characterised at 165 h after insemination according to the grading criteria of the International Embryo Transfer Society (IETS)^73^.

### Blastocyst fixation and staining

Blastocyst fixation (n = 164) and staining was performed in glass plates coated with Sigmacoate siliconizing reagent (Sigma-Aldrich, USA). For fixation, each blastocyst was washed once in 2% BSA/PBS and placed in 4% paraformaldehyde (PFA) for 12 mins at RT. Then the blastocysts were washed twice in 2% BSA/PBS for 5 mins. Subsequently they were placed in a 1% TritonX100 (Sigma-Aldrich, USA) solution for 3 min and washed twice in 2% BSA/PBS for 5 min at RT. Apoptosis was detected using the *In-Situ* Cell Death Detection Kit with TMR red (Roche, New Jersey, USA) following the manufacturer’s instructions. Specimens were then washed in 2% BSA/PBS and stained with Vectashield DAPI dye (Vector laboratories, USA) for the determination of total cell number. The blastocysts were then placed in individual micro wells of 10-well slides (ThermoFisher, UK), coverslipped and stored in the dark at 4 °C until fluorescence imaging.

### Evaluation of total cell number and apoptosis

For fluorescence microscopic analyses of the blastocysts, the Nikon Eclipse Ti 90x inverted microscope adjusted with a Hamamatsu digital camera (C4742-80-12AG) was used. Fluorescence filters (max. excitation wavelengths: 360 nm/DAPI, 540/fluorescein) were applied for evaluating the total cell number and the number of apoptotic cells. Each blastocyst was imaged in 7 focal planes using the Velocity 3D imaging software (PerkinElmer Inc., USA). The images were analysed and the total cell number, measured as number of nuclei in the embryonic cells, along with the number of apoptotic cells in each embryo were annotated using the Velocity 3D imaging software (PerkinElmer Inc., USA).

### Statistical analysis

Statistical significance of sperm motility assessments namely graded (A, B, C and D) motility, hyperactivation and morphokinetic parameters from different sexing treatments (SS or NS) was determined by paired t-tests, for dependent sample analyses. Fisher’s exact test was used to determine the embryo arrest at 2, 4 and 8 cell and blastocyst formation between sex-sorted sperm derived embryos compared to conventionally derived embryos. The cumulative arrest of embryos and the comparison of blastocyst development between sex-sorted and conventionally derived embryos on day 7 were analysed using the Chi-square test. The multivariate survival analysis with the Cox proportional-hazards regression was used to determine the relationship between fertilizing sperm origin and embryo survival and additionally to determine the relationship between individual bull and embryo survival time. Normal distribution was confirmed by the Kolmogorov-Smirnoff test. The comparison of the effect of the sperm type on the mean time to a specific embryonic stage was performed using the generalized mixed linear model. The chi square test was used to assess the rate of embryo shrinkage/fusion during development depending on fertilizing sperm origin. The Mann-Whitney U-test was carried out to determine developmental timings in relation to fertilizing sperm origin. The generalized estimating equation was used to determine the difference between total cell number and percentage of apoptotic cells in sexed as compared to conventionally derived embryos. All statistical analysis was conducted using IBM SPSS statistics v25 (SPSS Inc., Chicago, Ill., USA). For all analyses, p < 0.05 was considered statistically significant.

## Supplementary information

Supplementary information.

Supplementary Movie 1

Supplementary Movie 2

Supplementary Movie 3

Supplementary Movie 4

## Data Availability

The data that support the findings of this study are available from the corresponding author, Prof. Dr. Sabine Kölle, upon reasonable request.
