# Supplementary material for: Bovine Sperm Sexing Alters Sperm Morphokinetics and Subsequent Early Embryonic Development
Source: Sci Rep. 2020 Apr 10;10:6255. doi: 10.1038/s41598-020-63077-6 (PMC7148378; doi:10.1038/s41598-020-63077-6)
Supplement: Supplementary file 1 — Supplementary information. [file 41598_2020_63077_MOESM1_ESM.docx]

# Supplementary information

**Bovine Sperm Sexing Alters Sperm Morphokinetics and Subsequent Early Embryonic**

**Development**

H. Steele^1^, D. Makri^2^, W.E. Maalouf^2^, S. Reese^3^, and S. Kölle^1^ ^*^

^1^School of Medicine, Health Sciences Centre, University College Dublin, Dublin, Ireland

^2^School of Medicine, University of Nottingham, Medical School, Nottingham, United Kingdom

^3^School of Veterinary Medicine, Institute of Veterinary Anatomy, Histology and Embryology, University of Munich, Germany

^*^Correspondence: Sabine Kölle, School of Medicine, University College Dublin, Stillorgan Road, Belfield, Dublin 4, Ireland. Email: [sabine.koelle@ucd.ie](mailto:sabine.koelle@ucd.ie)

[heather.steele@ucdconnect.ie](mailto:heather.steele@ucdconnect.ie)

[dimikri@yahoo.gr](mailto:dimikri@yahoo.gr)

[walid.maalouf@nottingham.ac.uk](mailto:walid.maalouf@nottingham.ac.uk)

[s.reese@anat.vetmed.uni-muenchen.de](mailto:s.reese@anat.vetmed.uni-muenchen.de)

# Supplementary Table 1. Embryo development in vitro from oocytes fertilized with sexed or conventional sperm

| **Developmental stage** | **Fertilisation with conventional sperm N. (%)** | **Fertilisation with sexed sperm**  **N. (%)** |
| --- | --- | --- |
| Oocytes | 179 | 176 |
| 2-cell D2 | 160 (89.4) | 132 (75) |
| 4-cell D3 | 143 (79.8) | 118 (67) |
| 8-cell D4 | 114 (63.7) | 79 (44.8) |
| Blastocyst D7 | 20 (11.2) | 23 (13) |
| Expanding blastocyst D7 | 78 (43.6) | 45 (25.5) |
| Hatching blastocyst D7 | 12 (6.7) | 12 (6.8) |

**Legends for Supplemental Files**

**Supplementary Table 1:** Table 1. Embryo development in vitro from oocytes fertilized with sexed or conventional sperm

**Movie 1:** Full time-lapse videomicroscopy of a bovine unfertilized oocyte or an arrested zygote, failing to cleave and undergo further development on day 2 after insemination with sexed sperm.

**Movie 2:** Full time-lapse videomicroscopy development of a sexed sperm derived bovine embryo undergoing blastomere lysis.

**Movie 3:** Full time-lapse videomicroscopy development of a conventional sperm derived bovine embryo developing to a blastocyst.

**Movie 4:** Full time-lapse videomicroscopy development of a sexed sperm derived bovine embryo developing to a blastocyst.
